# Dissecting the Cell Entry Pathway of Dengue Virus by Single-Particle Tracking in Living Cells

**DOI:** 10.1371/journal.ppat.1000244

**Published:** 2008-12-19

**Authors:** Hilde M. van der Schaar, Michael J. Rust, Chen Chen, Heidi van der Ende-Metselaar, Jan Wilschut, Xiaowei Zhuang, Jolanda M. Smit

**Affiliations:** 1 Department of Medical Microbiology, Molecular Virology Section, University Medical Center Groningen, University of Groningen, Groningen, The Netherlands; 2 Department of Molecular and Cell Biology, Harvard University, Cambridge, Massachusetts, United States of America; 3 Department of Chemistry and Chemical Biology, Harvard University, Cambridge, Massachusetts, United States of America; 4 Department of Physics, Harvard University, Cambridge, Massachusetts, United States of America; 5 Howard Hughes Medical Institute, Harvard University, Cambridge, Massachusetts, United States of America; Harvard Medical School, United States of America

## Abstract

Dengue virus (DENV) is an enveloped RNA virus that causes the most common arthropod-borne infection worldwide. The mechanism by which DENV infects the host cell remains unclear. In this work, we used live-cell imaging and single-virus tracking to investigate the cell entry, endocytic trafficking, and fusion behavior of DENV. Simultaneous tracking of DENV particles and various endocytic markers revealed that DENV enters cells exclusively via clathrin-mediated endocytosis. The virus particles move along the cell surface in a diffusive manner before being captured by a pre-existing clathrin-coated pit. Upon clathrin-mediated entry, DENV particles are transported to Rab5-positive endosomes, which subsequently mature into late endosomes through acquisition of Rab7 and loss of Rab5. Fusion of the viral membrane with the endosomal membrane was primarily detected in late endosomal compartments.

## Author Summary

Dengue virus (DENV) is the most common arthropod-borne infection worldwide with 50–100 million cases annually. Despite its high clinical impact, little is known about the infectious cell entry pathway of the virus. Previous studies have shown conflicting evidence about whether the virus fuses directly with the cell plasma membrane or enters cells by receptor-mediated endocytosis. In this manuscript, we dissect the cell entry pathway of DENV by tracking single fluorescently labeled DENV particles in living cells expressing various fluorescent cellular markers, using real-time multi-color fluorescence microscopy. We show that DENV particles are delivered to pre-existing clathrin-coated pits by diffusion along the cell surface. Following clathrin-mediated uptake, the majority of DENV particles are transported to early endosomes, which mature into late endosomes, where membrane fusion occurs. This is the first study that describes the cell entry process of DENV at the single particle level and therefore provides unique mechanistic and kinetic insights into the route of entry, endocytic trafficking behavior, and membrane fusion properties of individual DENV particles in living cells. This paper opens new avenues in flavivirus biology and will lead toward a better understanding of the critical determinants in DENV infection.

## Introduction

Dengue virus (DENV) is a mosquito-transmitted, enveloped RNA virus that belongs to the family *Flaviviridae*. This family also includes West-Nile virus (WNV) and tick-borne encephalitis virus (TBEV). DENV causes the most common arthropod-borne infection worldwide with 50–100 million cases annually [1]–[3]. Despite its threat to human health, there are presently neither vaccines nor antiviral drugs to prevent or treat dengue infection. The development of novel therapies requires insight into the viral life cycle. A potential target for intervention strategies is the infectious cell entry pathway.

The infectious entry of DENV is mediated by the viral envelope glycoprotein E, which is organized in 90 homodimers on the surface of the virion [4],[5]. The E glycoprotein is involved in interaction with cellular receptors as well as the subsequent membrane fusion process [6]–[8]. *In vitro* studies with TBEV indicate that membrane fusion is triggered upon exposure of the virus to low pH [8]. At low pH, the E proteins undergo a dramatic re-organization which leads to the formation of E trimers [9]. The crystal structure of the E protein has been solved in its dimeric pre-fusion, and trimeric post-fusion configurations [10],[11]. Although much is known about the molecular mechanisms involved in the membrane fusion process, many critical questions regarding the cell entry pathway of flaviviruses remain unanswered.

The cell entry mechanism of DENV remains controversial. Early electron microscopy studies provided evidence for direct fusion with the plasma membrane [12],[13], whereas a recent study indicates that DENV enters cells via clathrin-mediated endocytosis [14]. Clathrin-mediated endocytosis involves internalization of ligands and receptors through a clathrin-coated pit, which buds into the cell cytosol and delivers its cargo to early endosomes and subsequently to late endosomes and lysosomes [15]–[17]. Other flaviviruses have also been described to infect their host cell via clathrin-mediated endocytosis [18]–[21]. Evidence for flavivirus entry via this pathway is based on the use of inhibitors of clathrin-mediated uptake, such as chlorpromazine and dominant-negative mutants of Eps15 [18],[20],[22]. Furthermore, addition of acidotropic reagents to cells has been observed to dramatically reduce viral infectivity and membrane fusion activity, suggesting that flaviviruses mediate membrane fusion from within acidic endosomes [23]–[26]. A recent study on the entry of WNV particles demonstrates that WNV colocalizes with the early endosome marker EEA-1 (Early Endosome Antigen-1), and at later time points with the late endosome/lysosome marker LAMP-1 (Lysosome-Associated Membrane Protein-1) [27]. Taken together, these studies suggest clathrin-mediated endocytosis as a viable pathway for flavivirus entry, but the exact manner in which DENV virus particles enter cells and traffic through the endocytic network remains unclear, as does the identity of the organelle in which viral fusion occurs.

In this study, we dissected the cell entry pathway of DENV by tracking fluorescently labeled DENV particles in living cells expressing various fluorescent cellular markers using real-time multi-color fluorescence microscopy. These experiments demonstrate that DENV infects its host cell via clathrin-mediated endocytosis. DENV particles move on the cell surface in a diffusive manner until they join a pre-existing clathrin-coated pit. Following clathrin-mediated uptake, the majority of DENV particles enter early endosomes that progress to late endosomes, where membrane fusion occurs.

## Results

### Dengue Virus Enters Cells via Clathrin-Mediated Endocytosis

**Figure 1:**
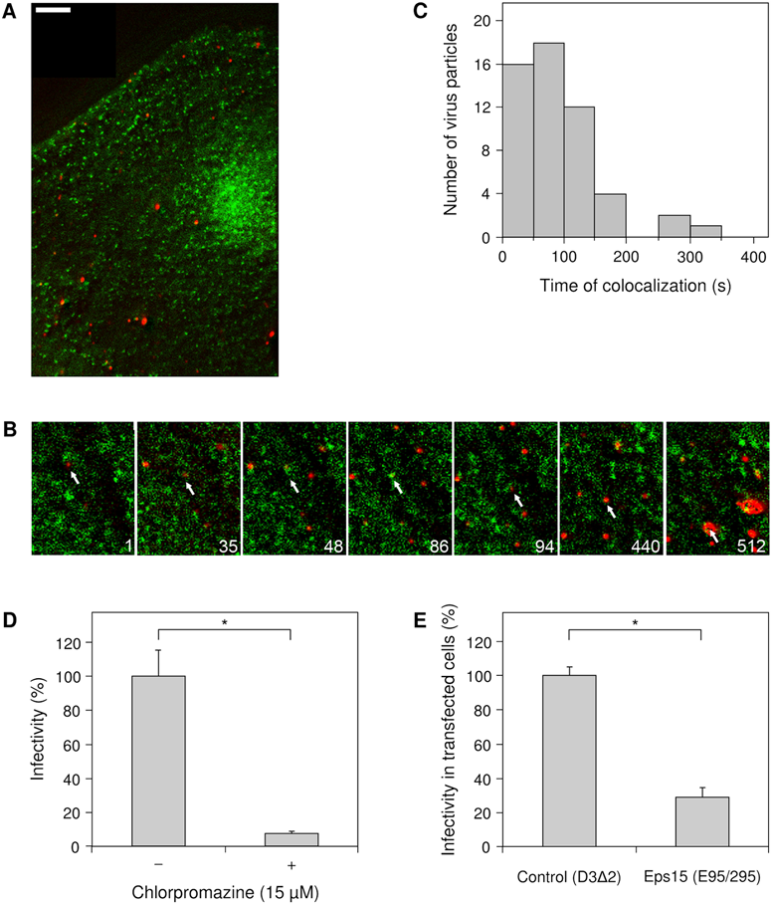
DENV enters cells via clathrin-mediated endocytosis. (A) Fluorescent image of a LCa-eYFP-expressing cell (green) infected with DiD-labeled DENV particles (red). Scale bar is 10 µm. (B) Selected frames from a single DENV particle (red), indicated with the arrow, entering a cell via clathrin-mediated endocytosis. The numbers designate seconds post-binding. (C) Histogram of the time that DENV particles colocalized with clathrin-eYFP. (D) DENV infectivity in BS-C-1 cells in the absence or presence of 15 µM chlorpromazine. Viral infectivity was measured at 24 hours post-infection by counting the number of E protein–expressing cells using immunofluorescence microscopy. The experiment was performed in triplicate, and the bars represent the average±SD. ^*^ *P*<0.01. (E) DENV infection in HeLa cells expressing a dominant-negative Eps15 mutant (E95/295) or its vector control (D3Δ2). At 30 hours post-transfection, HeLa cells were infected with DENV for 21 hours and subsequently stained for expression of E-proteins. Cells were analyzed by flow cytometry, and the results are expressed as the percentage infectivity in transfected cells. The experiment was performed in triplicate, and the bars represent the average±SD. ^*^ *P*<0.01.

In order to visualize single DENV particles in living cells, we labeled the virus with the lipophilic fluorescent probe DiD. The concentration of the DiD dye in the viral membrane is sufficiently high so that its fluorescence is largely quenched, but still allows single DiD-labeled virions to be detected. Membrane fusion can be observed as fluorescence dequenching. We have shown previously that this labeling procedure does not affect the infectious properties of DENV [26]. The tracking experiments were performed in African green monkey kidney cells (BS-C-1), which are highly permissive to DENV infection [26],[28]. To test whether DENV is internalized through clathrin-mediated endocytosis, BS-C-1 cells stably expressing enhanced yellow fluorescent protein (eYFP) fused to the light chain of clathrin (LCa-eYFP) were used. We and others have previously shown that LCa-eYFP highlights more than 95% of the coated pits and vesicles in living cells and that this fusion protein does not disturb the functional integrity of clathrin molecules [29],[30]. DiD-labeled DENV was added *in situ* to these cells at 37°C and fluorescent images were recorded at 2 frames per second for 25 min. Figure 1A shows the distribution of LCa-eYFP (green) and DiD-labeled DENV particles (red) in a cell. The LCa-eYFP signal appeared as discrete structures in cells. A typical example of a DENV entry event is shown in Figure 1B. In this example, the virus particle first binds to and moves along the cell surface. Forty-eight seconds post-binding, the virus particle associates with a discrete spot containing the LCa-eYFP signal. Thereafter, the clathrin signal around the virus particle increases, indicating maturation towards a clathrin-coated vesicle. At 94 seconds, the clathrin signal rapidly disappears, presumably due to uncoating of the clathrin-coated vesicle. Membrane fusion eventually occurs at 512 seconds post-infection. Quantitative analysis of 47 virus trajectories revealed that 98% of the DENV particles that fused with endosomes entered through LCa-eYFP positive clathrin-coated pits. On average, the clathrin signal colocalized with the virus particle for 83 seconds (Figure 1C), which is consistent with previously observed dynamics of clathrin-mediated endocytosis [30],[31].

To confirm that DENV specifically enters cells via clathrin-mediated endocytosis, we investigated the effects of chlorpromazine, a cationic amphiphilic drug that inhibits the formation of clathrin-coated pits [32], and of a dominant-negative mutant form of Eps15 (E95/295), a protein required for clathrin-dependent uptake [33], on DENV infectivity. Viral infectivity was severely impaired in cells treated with chlorpromazine (Figure 1D) and significantly reduced in cells expressing dominant-negative Eps15 (Figure 1E). Furthermore, no membrane fusion events were seen in real-time virus tracking experiments in chlorpromazine-treated cells (results not shown). Taken together, these results indicate that DENV requires clathrin-mediated endocytosis for its infectious entry.

### Dengue Virions Are Delivered to Pre-Existing Clathrin-Coated Pits by Diffusion

**Figure 2:**
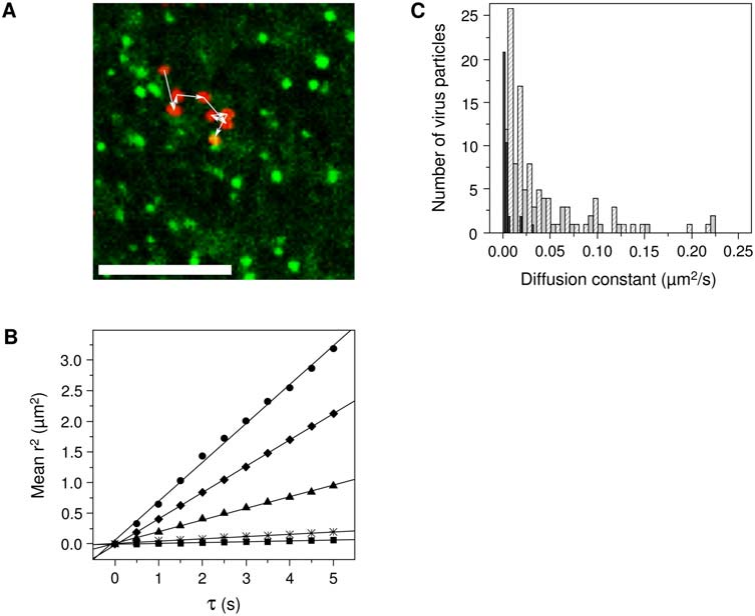
DENV diffuses along the cell surface and joins a pre-existing clathrin-coated pit. (A) Overlay of a series of time-lapsed images of a virus particle prior to entering a pre-existing clathrin-coated pit. Each depicted virus image (red) is taken 4 seconds apart, and the positions are connected in time with white arrows. For clarity, the LCa-eYFP signal (green) was averaged over 10 seconds, starting at the moment when the virus first overlaps the clathrin-coated pit. Scale bar is 10 µm. (B) MSD-plot for 5 example virus trajectories prior to association with clathrin-coated pits. The diffusion constants are 0.003 µm^2^/s (squares), 0.009 µm^2^/s (asterisks), 0.047 µm^2^/s (triangles), 0.107 µm^2^/s (diamonds), and 0.159 µm^2^/s (circles). (C) Diffusion constants of virus particles prior to entrance into a clathrin-coated pit (light gray bars), during colocalization with clathrin (black bars), and on cells treated with chlorpromazine (dashed bars).

Tracking individual particles also allowed us to determine how DENV particles recruit clathrin-coated pits. A detailed characterization of the individual trajectories showed that virus particles associate with clathrin on average at 111 s post-attachment to the cell surface. Nearly all particles (92%) were observed to move along the cell surface and join a pre-existing clathrin-coated pit. The remaining minor fraction either appeared to land directly on a pre-existing clathrin-coated pit or a clathrin-coated pit was formed directly at the site of the virus particle. An example of the surface motion of DENV towards a clathrin-coated pit is depicted in Figure 2A and Video S1, which is published as supporting information on the web site.

Subsequently, we investigated whether the surface motion of DENV is characterized by random diffusion or directed movement. To this end, we plotted the mean-square displacement (MSD) of each particle prior to association with a clathrin-coated pit as a function of time. A linear relationship between MSD and time would indicate simple diffusion, an upward curvature designates directed motion, and a downward curvature implies diffusion within a confined region. Figure 2B gives the MSD plot for 5 typical virus trajectories. The apparent linear relationship between the MSD and time indicates that DENV moved on the cell surface in a diffusive manner. During the tracking experiments, we noticed that the mobility of the virus drops when the particle overlaps with a clathrin-coated pit. To obtain a quantitative insight into this behavior, we calculated the diffusion constants from the MSD plots for each particle prior to or during colocalization with clathrin and used that as a measure for surface mobility of the virus. The results show that many DENV particles that were associated with the cell surface were quite mobile, but once they were captured by a clathrin-coated pit their mobility was highly reduced (Figure 2C). Furthermore, treatment of cells with chlorpromazine revealed that DENV particles remained migrating along the cell surface throughout the duration of the experiment in a manner similar to that seen for particles prior to clathrin-mediated entry in untreated cells (Figure 2C).

### Endocytic Trafficking of Dengue Virus Particles

Following clathrin-mediated internalization, virus particles are typically trafficked along an endocytic pathway, which comprises a network of highly dynamic vesicles and endosomes. Endocytic trafficking is regulated by a large family of small Rab GTPases [34]–[38]. Specific Rab GTPases are often enriched in distinct intracellular vesicles and may be used to identify endocytic vesicles and endosomes. For example, Rab5 and Rab7 primarily decorate early and late endosomes, respectively [39]–[41]. Recent live-cell imaging studies have also revealed a small fraction of the endosomes containing both Rab5 and Rab7, which likely indicates intermediate endosomes that are maturing towards late endosomes [34],[35],[37].

**Figure 3:**
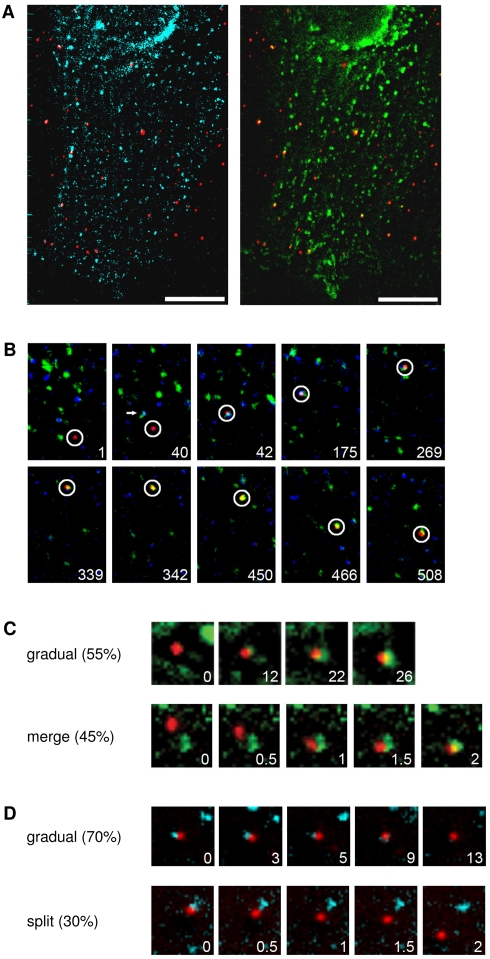
DENV particles are transported to early endosomes that mature into late endosomes. (A) Fluorescent images of a cell expressing Rab5-eCFP (light blue) and Rab7-eYFP (green) upon infection with DiD-labeled DENV (red). Scale bar is 10 µm. (B) The selected frames show the endocytic trafficking behavior of a single DiD-labeled DENV particle (surrounded by a white circle) in a cell. The numbers designate seconds post-infection. (C) Snapshots of a virus trajectory showing the modes of Rab7 accumulation. At all time frames, the virus particle colocalizes with Rab5, but for clarity the signal is not depicted. Two modes of Rab7 accumulation were observed: a gradual recruitment of Rab7 (upper panel) or merging of the endosome with an existing Rab7-positive endosome (lower panel) was observed. The numbers indicate the time frame (in seconds) of endosome maturation. (D) Snapshots of a virus showing the modes of Rab5 exit. At all time frames, the virus particle colocalizes with Rab7, but for clarity the signal is not depicted. The exit of Rab5 signal also appears to take place in two different modes: a gradual release of Rab5 (upper panel) or splitting off a Rab5-containing endosome (lower panel).

To study the itinerary of endosomal compartments visited by DENV, we tracked single DiD-labeled virus particles in BS-C-1 cells co-expressing Rab5 fused to enhanced cyan fluorescent protein (Rab5-eCFP) and Rab7 fused to enhanced yellow fluorescent protein (Rab7-eYFP). We have used this approach before and observed that low level expression of Rab5-eCFP and Rab7-eYFP in cells does not adversely affect endocytic trafficking inside the cell [34]. A typical example of a cell co-transfected with Rab5-eCFP and Rab7-eYFP early after infection with DiD-labeled DENV particles is depicted in Figure 3A. Rab5- and Rab7-positive endosomes can be observed as clear distinct spots that are localized in the cell periphery as well as in the perinuclear region of the cell. We analyzed 51 virus trajectories in total and observed that 86% of the particles first enter Rab5-positive early endosomes; the other 14% of the virions are directly delivered to Rab5/Rab7-positive intermediate endosomes. An example of DENV endocytic trafficking is shown in Figure 3B and Video S2, which is published as supporting information on the web site. At 42 seconds post-binding, this particular virus particle moves with a velocity of 0.42 µm/s towards an intermediate endosome, as shown by the colocalization with Rab5-eCFP and Rab7-eYFP. Subsequently, the intermediate endosome enclosing the virus particle matures into a late endosome as detected by the disappearance of the Rab5 signal. At 342 seconds, the virus particle resides in a Rab7-positive late endosome and induces membrane fusion from within this organelle at 508 seconds post-infection as detected by a five-fold increase in the DiD-intensity. An example of DENV intracellular trafficking via Rab5-positive endosomes is shown in Video S3, which is published as supporting information on the website.

We observed different modes of Rab7 accumulation and Rab5 dissociation during endosome maturation. Rab5-positive early endosomes carrying DENV particles were found to mature either through a gradual appearance of Rab7 (55%) or by merging with an existing Rab7-positive endosome (45%). Typical examples of these modes of Rab7 accumulation are depicted in Figure 3C, and Videos S4 and S5. Likewise, the exit of the Rab5 signal also appears to take place in different modes. About 70% of the intermediate endosomes complete the maturation process by a gradual release of Rab5 (Figure 3D, Video S6). In the remaining cases, the virus particle appeared to be sequestered into a Rab7-enriched domain of the intermediate endosome, which subsequently pinched off and moved away as a late endosomal compartment (Figure 3D, Video S7).

We have previously identified two distinct populations of Rab5-positive early endosomes [34]. A group of dynamic early endosomes are transported on microtubules and rapidly mature towards late endosomes, while the remaining are relatively static and mature much more slowly. Influenza virus, low density lipoproteins, and epidermal growth factors were previously found to be preferentially targeted to the dynamic, rapidly maturing population, whereas transferrin is non-selectively delivered to both populations. DENV was non-selectively delivered to both endosome populations (data not shown).

### Dengue Virus Fuses Primarily from within Rab7-Positive Late Endosomes

**Figure 4:**
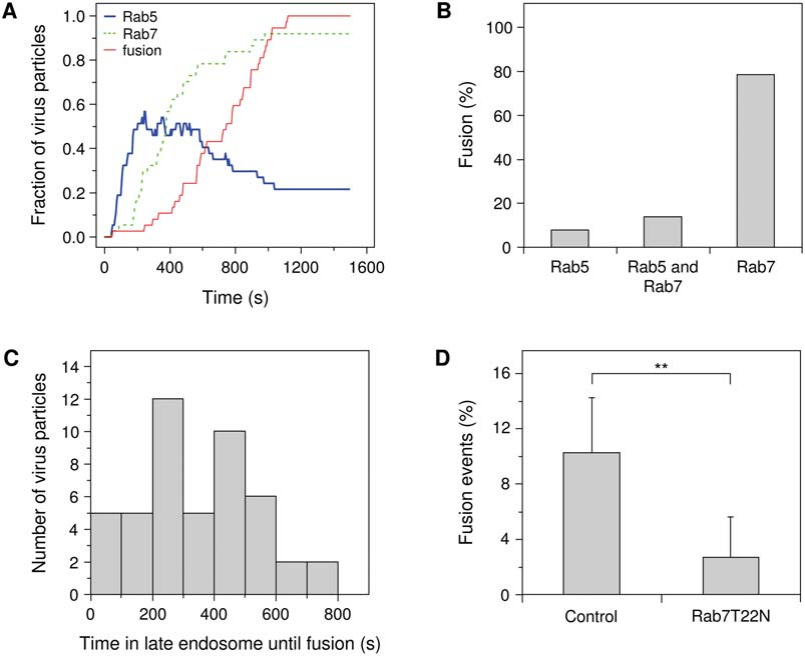
DENV fuses primarily from within late endosomes. (A) Individual DENV trajectories were aligned to the time of binding to the cell surface. Fraction of virus particles that colocalize with Rab5 and Rab7, and that undergo membrane fusion are shown as a function of time. (B) Quantification of the percentage of membrane fusion events in the different endocytic compartments. (C) Histogram of the time that virus particles spend in Rab7-positive late endosomes prior to the onset of membrane fusion. (D) The number of DENV membrane fusion events in BS-C-1 cells transfected with Rab7T22N or with a control plasmid. The experiment was carried out more than 10 times and the bars represent the mean±SD. ^**^ *P*<0.001.

Enveloped viruses escape from the endocytic pathway by a membrane fusion reaction. In our experimental set-up, membrane fusion can be detected as a sudden increase of DiD fluorescence due to the dilution of the DiD-probes from the viral membrane into the endosomal membrane. This assay allowed us to directly examine the nature of the endosomes from which DENV mediates membrane fusion. Individual virus trajectories showed that the majority of the virus particles first joined a Rab5-positive endosome, which then matured through the Rab5/Rab7-copositive intermediate stage into a Rab7-positive late endosome, were membrane fusion was observed. Figure 4A gives a quantitative kinetic analysis of the endocytic trafficking behavior and membrane fusion events of all analyzed DENV particles. Internalization of DENV particles appeared to be relatively quick, since 50% of the particles localized to early endosomes at 3.5 minutes post-attachment to the cell surface. Thereafter, the particles started to associate with Rab7-positive endosomes. The first membrane fusion events were detected at 5 minutes post-infection, and nearly all fusion events occurred within 17 minutes post-infection. The average time point of membrane fusion was 12.5 min, which is in agreement with our previous results [26].

The vast majority (80%) of particles induced membrane fusion from within Rab7-positive late endosomes devoid of any detectable Rab5 signal, while 15% of the particles fused from within Rab5/Rab7-copositive intermediate endosomes. Only 5% of the virus particles fused from within Rab5-positive early endosomes that lack Rab7 (Figure 4B). Membrane fusion was initiated at on average 5.5 minutes post-entry of DENV into the Rab7-positive endosomes (Figure 4C).

Our observation that DENV (serotype 2, strain S1) primarily fuses from within Rab7-positive late endosomes is somewhat surprising, since a recent report showed that expression of dominant-negative Rab7T22N did not affect DENV (serotype 2, strain New Guinea C) infectivity [22]. To investigate whether this discrepancy is related to the different virus strains used, we analyzed the infectious properties of both viruses on HeLa cells expressing dominant-negative Rab5S34N and Rab7T22N. In agreement with the above results, viral infectivity of S1 was severely impaired in cells expressing dominant-negative Rab7, whereas the infectious properties of NGC were unaffected under the conditions of the experiments (Figure S1). To investigate the requirement for Rab7 of S1 infectivity in more detail, we performed single-particle tracking experiments in cells transiently expressing the dominant-negative Rab7T22N mutant [42],[43]. Figure 4D shows that the number of membrane fusion events was significantly reduced by a factor of 4 in these cells (T-test: *P*<0.001), which indicates that S1 needs to travel to Rab7-positive endosomes to undergo membrane fusion.

## Discussion

Despite the medical importance of DENV, little information is available about the infectious cell entry pathway of the virus. In this study, we investigated the cell entry process of single DENV particles in real-time by simultaneous tracking of fluorescently labeled DENV particles and endocytic structures in cells. This approach allowed us to obtain mechanistic and kinetic insights into the route of internalization and endocytic trafficking behavior of individual DENV particles in living cells.

Previous electron-microscopy studies suggested that DENV penetrates both mammalian and insect cells by direct fusion with the plasma membrane [12],[13]. In contrast, this report shows that DENV enters cells via clathrin-mediated endocytosis and fuses from within late endosomes. We observed that more than 98% of the particles that underwent membrane fusion, first associated with a clathrin-coated structure for a substantial time period. Furthermore, treatment of cells with chlorpromazine as well as expression of a dominant-negative Eps15 mutant significantly suppressed the number of DENV-infected cells. It is not clear what the explanation is for the discrepancy, but it might be related to the methodology that was used to investigate the cell entry process of the virus. The conclusion that DENV utilizes clathrin-mediated endocytosis for internalization is in agreement with recent observations of Acosta and co-workers [14]. During the course of this study, these investigators published that DENV infectivity in C6/36 mosquito cells is severely inhibited after treatment of the cells with a variety of chemical and molecular inhibitors of clathrin-mediated endocytosis.

Real-time imaging studies showed that macromolecules either induce *de novo* formation of clathrin-coated pits or are recruited to pre-existing clathrin-coated pits [31],[44],[45]. For example, influenza virus particles land on the cell surface and induce *de novo* formation of clathrin-coated pits at the site of binding [30]. This study indicates that DENV particles first diffuse along the cell surface before they encounter pre-existing clathrin-coated pits. After the virus associates with the pit, the clathrin signal around the virus particle increases, which implies maturation of the clathrin-coated pit and formation of a clathrin-coated vesicle. Thereafter, the clathrin signal rapidly disappears again, typically within a time scale of a few seconds. This behavior is similar to that of reoviruses, which have been shown to stabilize and induce maturation of pre-existing clathrin-coated pits [31].

Recently, several modes of endosome maturation have been described. Rink et al. showed that Rab5-positive vesicles, which have split off from a dynamic early endosomal network, accumulate Rab7 and subsequently gradually lose Rab5 [35]. Vonderheit et al. found that Rab5-positive endosomes, containing Semliki Forest Virus (SFV) particles, gradually acquire Rab7 in a separate domain. The SFV particles are sequestered into this Rab7 domain, which pinches off as a Rab7-positive late endosome, leaving a Rab5-positive endosome behind [37]. We observed both modes of endosome maturation. Most DENV particles progressed from early to late endosomes by gradual appearance of Rab7 and a gradual loss of Rab5. In addition, 45% of Rab5-positive endosomes carrying DENV merged with existing Rab7-positive endosomes. Occasionally, we observed that DENV particles sequestered into a distinct Rab7 domain, similar to the behavior observed from endosomes containing SFV [37].

DENV particles predominantly fused from within Rab7-positive endosomes. Furthermore, the membrane fusion activity was significantly impaired in cells expressing dominant-negative forms of Rab7, which indicates that progression of DENV to Rab7-positive endosomes is important for its infectious entry. In contrast, Krishnan et al. have recently demonstrated that the infectivity of DENV-2 strain NGC was not affected by dominant-negative Rab7, while ablation of Rab5 severely reduced the number of infected cells [22]. A direct comparison between both virus strains revealed that viral infectivity of S1 was severely impaired in cells expressing dominant-negative Rab7, whereas the infectivity of NGC was unaffected. These results suggest that both virus strains have distinct entry characteristics. In this respect it is interesting to note that DENV-2 strain NGC induces syncytium formation in a fusion from without assay at pH 6.4, whereas the pH threshold for the DENV-2 S1 strain is around pH 5.8 (personal communication, Dr. P. Young, University of Queensland, Australia). The different pH-dependent properties of these virus strains may therefore reflect the distinct requirements for functional endocytic trafficking in cells. Future experiments should reveal whether the pH threshold determines in which organelle membrane fusion occurs.

DENV particles reside on average for 5.5 min in Rab7-positive endosomes prior to the onset of membrane fusion. This result is surprising considering that TBEV efficiently fuses with liposomes in a model system in a time scale of seconds after low-pH exposure [46]. Pre-exposure of TBEV to low pH for 10–20 seconds in the absence of liposomes completely abolishes the membrane fusion activity of the virus [46]. Similar results were obtained for WNV (unpublished results, J. Wilschut and J. M. Smit). Our finding that DENV fuses several minutes after entering a late endosome might therefore suggest that, in addition to exposure to the acidic lumen of the late endosome, other cellular factors are involved in the activation of the membrane fusion machinery of DENV. Another possibility is that the accumulation of Rab7 significantly precedes acidification to the fusion pH.

Taken together, we propose the following model for cell entry of DENV S1 strain. First, the virus particle binds to a cellular receptor. Subsequently, DENV diffuses as a virus-receptor complex or rolls over multiple receptors along the cell surface towards a clathrin-coated pit. Upon capture by a pre-existing clathrin-coated pit, the virus particles loses its mobility. Then, the clathrin-coated pit matures and pinches off into the cell cytoplasm to deliver the particles to Rab5-positive early endosomes. In general, the early endosome carrying the virus matures into a late endosome by gradual accumulation of Rab7, followed by a gradual loss of Rab5. Finally, the DENV particles localize to Rab7-positive late endosomes and move through the cytoplasm of the cell until the onset of membrane fusion allows the genetic material of the virus to be delivered into the cytoplasm.

Single-particle tracking has substantially enriched our knowledge on viral cell entry mechanisms and has revealed previously unknown aspects of virus-host interactions [30],[47],[48]. The mechanistic and kinetic insights offered by this technique provide a better understanding of disease pathogenesis and may lead to a rational design of antiviral drugs and vaccines. This is the first study that describes the cell entry pathway of DENV at a single-particle level. The parameters obtained in this study will serve as a framework for our current study on the fate of individual antibody-opsonized DENV particles into Fc receptor-bearing to elucidate the molecular basis of antibody-dependent enhancement of DENV infection.

## Materials and Methods

### Cells

*Aedes albopictus* C6/36 cells were maintained in Minimal Essential Medium (MEM; Life Technologies, Breda, The Netherlands) supplemented with 10% fetal bovine serum, 25 mM HEPES, 7.5% sodium bicarbonate, 200 mM glutamine, 100 µM non-essential amino acids, penicillin (100 U/ml), and streptomycin (100 µg/ml) at 30°C, 5% CO_2_. HeLa cells were cultured in a 1∶1 mix of DMEM (Life Technologies) and HAM (Life Technologies) supplemented with 10% fetal bovine serum, 25 mM HEPES, penicillin (100 U/ml), and streptomycin (100 µg/ml) at 37°C, 5% CO_2_. BS-C-1 cells were maintained in MEM (Invitrogen, Carlsbad, CA, USA) supplemented with 10% fetal bovine serum at 37°C, 5% CO_2_. BS-C-1 cells stably expressing LCa-YFP were created by use of the RetroMax retroviral expression system (Imgenex, San Diego, CA, USA) and cultured in BS-C-1 medium [30]. BS-C-1 cells were grown on glass coverslips (MatTek, Ashland, MA, USA), and prior to the tracking experiments washed with serum-free, phenol red-free medium.

### DNA Transfection of Cells

The plasmid encoding Rab5-eCFP was a gift from Dr. M. Zerial (Max Planck Institute, Dresden, Germany). The Rab7-eYFP plasmid was previously constructed by Dr. M. Lakadamyali [34]. The GFP-tagged dominant-negative Eps15 mutant E95/295 and its empty vector D3Δ2 were kindly provided by Dr. A. Benmerah and Dr. A. Dautry-Varsat (Institute Pasteur, Paris, France). The plasmids encoding GFP-tagged dominant-negative Rab5 mutant Rab5S34N, wild-type Rab5-GFP, myc-tagged dominant-negative Rab7 mutant Rab7T22N, and wild-type Rab7-myc were gifts from Dr. P. van der Sluijs (University Medical Center, Utrecht, The Netherlands). Cells were transfected with the plasmids using the transfection reagent FuGENE, according to the manufacturer's protocol (Roche, Nutley, NJ, USA).

### Viral Infectivity

To analyze the route of DENV cell entry, viral infectivity was measured in HeLa cells expressing dominant-negative Eps15 mutants. At 30 hours post-transfection, cells were infected at MOI 5. At 21 hours post-infection, cells were washed with PBS, trypsinized, fixed with 4% paraformaldehyde, and permeabilized with 0.5% saponin in PBS containing 2% FBS. Expression of the myc-tagged plasmids was detected with monoclonal antibody A-14 (Santa Cruz Biotechnology, Santa Cruz, CA, USA). DENV infection was measured using the monoclonal antibody MAB8702 directed against the viral E protein (Chemicon, Hampshire, United Kingdom). Cells were analyzed on a FACS Calibur cytometer. The effect of chlorpromazine on DENV infectivity was determined by an infectious center assay in BS-C-1 cells as described before [25]. Chlorpromazine (15 µM) was added to the cells 30 min prior to addition of the virus. At 1 hour post-infection, cells were washed and fresh medium containing 20 mM ammonium chloride was added. At 24 hours post-infection, cells were fixed and stained intracellularly with MAB8702 to measure infection [26].

### Preparation of DiD-Labeled Virus

DENV serotype 2 strain PR159 S1, generously provided by Dr. Richard Kuhn (Purdue University, Lafayette, IN, USA), was produced and labeled with DiD as described previously [26]. Briefly, monolayers of C6/36 cells were inoculated with DENV at MOI 0.1. At 72 hours post-infection, the progeny virions were harvested, purified by ultracentrifugation, and cleared from tartrate using 100 kD filter devices (Millipore, Amsterdam, The Netherlands). Subsequently, 2 nmol DiD (Molecular Probes, Eugene, OR, USA) dissolved in dimethyl sulfoxide (DMSO) was mixed with approximately 5×10^9^ genome-containing DENV particles while vortexing in a total DMSO concentration of less than 2.5%. After 10 min, the unincorporated dye was removed by gel filtration. DiD-labeled virus was stored at 4°C and used within 2 days. Virus preparations were analyzed with respect to the infectious titer and the number of physical particles, as described previously [26].

### Live-Cell Imaging

Tracking experiments were carried out 24 to 48 hours post-transfection as described previously [34]. Briefly, fluorescent images were recorded by exciting CFP with a 454 nm Argon laser (Melles-Griot, Carlsbad, CA, USA), YFP with a 532 nm Nd∶YAG laser (Crystalaser, Reno, NV, USA), and DiD with a 633 nm helium-neon laser (Melles-Griot). For the clathrin experiments, simultaneous images were recorded of DiD-labeled virions and LCa-eYFP at 2 frames per second. In case of the Rab5/Rab7 experiments, the excitation of DiD was continuous, whereas the excitation of CFP and YFP were alternated at 0.5 Hz. The fluorescent emission was spectrally separated by 650 nm long-pass dichroic mirrors (Chroma, Rockingham, VT, USA) and imaged onto two separate areas of charge-coupled device camera (CoolSNAP HQ, Roper Scientific). A 665 nm long-pass filter was used for the emission of DiD. For the emission of CFP and YFP, bandpass filters of 480/40 nm and 585/35 nm were used, respectively. The CFP and YFP filters were toggled by a motorized wheel at 0.5 Hz synchronically with the 454 nm and 532 nm lasers. Image analysis and single-particle tracking was performed using custom-written IDL software as described previously [26],[49]. Briefly, background and noise were reduced by convolution with a Gaussian spatial filter. Viral trajectories were generated by pairing virus spots in each frame according to proximity and similarity in intensity. Colocalization of viruses with fluorescent cellular markers was identified with an automated program and confirmed by eye, the criteria for colocalization being that the objects move together and have at least partial overlap. Only those particles that moved roughly within the focal plane and showed more than a fivefold increase in fluorescence intensity after membrane fusion were used for image analysis. Characterization of the movement of DENV particles on the cell surface was done by generating MSD-plots. The MSD at time interval τ is the average of all squared displacements throughout the virus trajectory prior to or during association with clathrin. The diffusion constants were calculated from the slope of the MSD-plot.

## Supporting Information

Figure S1 Effect of functional repression of Rab5 or Rab7 on the infectivity of DENV S1 strain and NGC strain. DENV S1 (light gray bars) and NGC infectivity (black bars) in HeLa cells expressing either wild-type Rab5 and Rab7 or dominant-negative Rab5 (Rab5S34N) and Rab7 (Rab7T22N). At 30 hours post-transfection, HeLa cells were infected with DENV for 21 hours and subsequently stained for E protein-expression. Cells were analyzed by flow cytometry, and the results are expressed as the percentage infectivity in transfected cells. The experiment was performed in triplicate, and the bars represent the average±SD.
(0.39 MB TIF)

Video S1 Surface motion of a DENV particle before capture by a clathrin-coated pit. DENV particle (red, surrounded by a white circle) that moves along the cell surface until it associates with clathrin (green). Upon binding, the clathrin intensity increases around the virus particle, indicating the maturation of the clathrin-coated pit. Subsequently, the clathrin coat disappears and the particle exhibits a rapid movement towards the perinuclear region of the cell. Snapshots of this video are shown in Figure 2A. The playback speed is 15× real-time.
(8.35 MB AVI)

Video S2 Endocytic trafficking of DENV. Upon cell entry, the DENV particle (red, surrounded by a white circle) moves rapidly towards an intermediate endosome enriched in Rab5 (blue) and Rab7 (green). This moment is clarified by the appearance of “(A)”, the arrow points at the intermediate endosome. Subsequently, the intermediate endosome carrying the virus particle moves around and merges with other endosomes (indicated by the arrows): a Rab5-positive endosome (B) and an intermediate endosome (C). Next, the intermediate endosome carrying the virus matures into a late endosome through a gradual disappearance of Rab5 (D). Membrane fusion occurs from within the Rab7-positive endosome (E). Snapshots of this video are shown in Figure 3B. During real-time imaging, DiD-labeled DENV particles were recorded at 2 Hz, whereas the excitation of Rab5-eCFP and Rab7-eYFP were alternated at 0.5 Hz. Due to this recording scheme, the CFP and YFP frames are updated every 4 frames in the video, whereas the DiD signal is updated every frame, which might give the impression that the endosomal markers are lagging behind the virus particle. The hallmarks of colocalization are that the DiD spot and the Rab signal at least show partial overlap when they are simultaneously excited, and that both signals are linked and move together in time. The playback speed is 10× real-time.
(9.66 MB AVI)

Video S3 Endocytic trafficking of DENV. DENV particle (red, surrounded by a white circle) that rapidly moves to a Rab5-positive early endosome (blue) on time point (A). The early endosome carrying the DENV particle acquires Rab7 (green) by merging with an intermediate Rab5- and Rab7-positive endosome (B). Subsequently, the endosome gradually accumulates more Rab7 (C) and gradually loses Rab5 (D). After merging of another large late endosome (E), the virus finally fuses from within the Rab7-positive late endosome (F). Real-time tracking was performed as described in the legend of Video S2. The playback speed is 10× real-time.
(10.20 MB AVI)

Video S4 Some early endosomes gradually accumulate Rab7 molecules. Endosome enclosing a DENV particle (red, surrounded by a white circle) that shows a gradual increase in the Rab7 signal (green). Although during real-time imaging DiD-labeled particles were excited at 2 Hz and Rab7-eYFP at 0.5 Hz, this video is composed of only those frames in which DiD and YFP were excited simultaneously. During the whole video, the virus particle colocalizes with Rab5, but for clarity the signal is not depicted. Snapshots of the maturation event are shown in Figure 3C. The playback speed is 10× real-time.
(8.00 MB AVI)

Video S5 Some early endosomes accumulate Rab7 by joining a pre-existing Rab7-positive endosome. Endosome enclosing a DENV particle (red, surrounded by a white circle) that merges with a Rab7-positive endosome (green). The arrow indicates the moment of the merging. Real-time imaging and the preparation of the video are described in the legends of Videos S2 and S4, respectively. During the whole video, the virus particle colocalizes with Rab5, but for clarity the signal is not shown. Snapshots of the maturation event are shown in Figure 3C. The playback speed is 10× real-time.
(4.43 MB AVI)

Video S6 Some endosomes complete the maturation process by a gradual release of Rab5 molecules. Endosome enclosing a DENV particle (red, surrounded by a white circle) that shows a gradual release of the Rab5 signal (blue). Real-time imaging was performed as described in the legend to Video S2, but only those frames in which DiD and CFP were excited simultaneously were used in the video. Furthermore, the virus particle colocalizes with Rab7 throughout the time trace, but for clarity the signal is not depicted. Snapshots of the maturation event are shown in Figure 3D. The playback speed is 10× real-time.
(5.97 MB AVI)

Video S7 Some endosomes complete the maturation process by splitting off a Rab5-positive endosome. Endosome enclosing a DENV particle (red, surrounded by a white circle) that splits off and leaves the Rab5-positive endosome (blue). The moment of splitting is indicated by the arrow, which points at the Rab5 endosome that is left behind. Real-time imaging and the preparation of the video are described in the legends to Videos S2 and S6, respectively. During the time trace, the virus particle colocalizes with Rab7, but for clarity the signal is not depicted. Snapshots of the maturation event are shown in Figure 3D. The playback speed is 10× real-time.
(2.93 MB AVI)
