# Supplementary figures and images for: Dissecting the Cell Entry Pathway of Dengue Virus by Single-Particle Tracking in Living Cells
Source: PLoS Pathog. 2008 Dec 19;4(12):e1000244. doi: 10.1371/journal.ppat.1000244 (PMC2592694; doi:10.1371/journal.ppat.1000244)

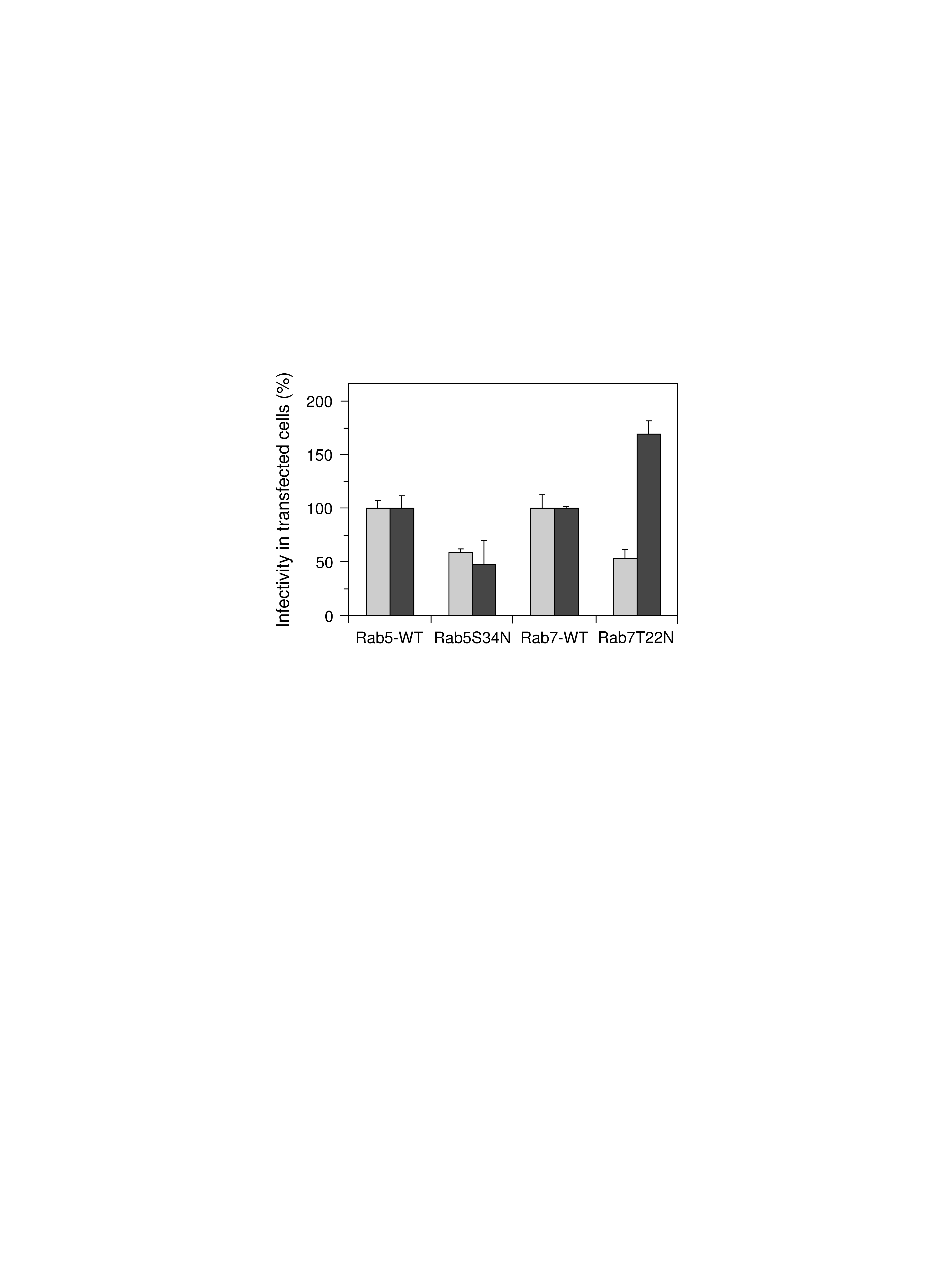

Supplement: Figure S1 — Effect of functional repression of Rab5 or Rab7 on the infectivity of DENV S1 strain and NGC strain. DENV S1 (light gray bars) and NGC infectivity (black bars) in HeLa cells expressing either wild-type Rab5 and Rab7 or dominant-negative Rab5 (Rab5S34N) and Rab7 (Rab7T22N). At 30 hours post-transfection, HeLa cells were infected with DENV for 21 hours and subsequently stained for E protein-expression. Cells were analyzed by flow cytometry, and the results are expressed as the percentage infectivity in transfected cells. The experiment was performed in triplicate, and the bars represent the average±SD. (0.39 MB TIF) [file ppat.1000244.s001.tif]
